# Abscisic Acid Can Play a Dual Role in the *Triticum aestivum*–*Stagonospora nodorum* Pathosystem

**DOI:** 10.3390/plants14030355

**Published:** 2025-01-24

**Authors:** Svetlana Veselova, Tatyana Nuzhnaya, Guzel Burkhanova, Sergey Rumyantsev, Igor Maksimov

**Affiliations:** 1 Institute of Biochemistry and Genetics, Ufa Federal Research Centre, Russian Academy of Sciences, Prospekt Oktyabrya, 71, 450054 Ufa, Russia; tanyawww89@mail.ru (T.N.); guzel_mur@mail.ru (G.B.); rumyantsev-serg@mail.ru (S.R.); maksimov@ufaras.ru (I.M.); 2 Ufa Institute of Biology, Ufa Federal Research Centre, Russian Academy of Sciences, Prospekt Oktyabrya, 69, 450054 Ufa, Russia

**Keywords:** abscisic acid, hormonal signaling pathways, necrotrophic effectors, SnToxA, SnTox3, defense response, redox status, catalase

## Abstract

Abscisic acid (ABA) is not only important for plant responses to abiotic stresses, but also plays a key and multifaceted role in plant immunity. In this work, we analyzed the role of ABA in the development of resistance/susceptibility in the wheat (*Triticum aestivum* L.)–*Stagonospora nodorum* Berk. pathosystem, which includes the recognition of the necrotic effectors (NEs) of a pathogen by the corresponding wheat susceptibility genes. We studied the interaction of the *S. nodorum* SnB isolate, which produces two NEs, SnToxA and SnTox3, with three wheat genotypes having different combinations of the corresponding host susceptibility genes (*Tsn1* and *Snn3-B1*). The results of this work on the gene expression and redox status of resistant and sensitive wheat genotypes treated with ABA show that ABA signaling is directed at inducing the resistance of wheat plants to *S. nodorum* SnB isolate through the activation of the early post-invasive defense genes *TaERD15* and *TaABI5.* The induction of the expression of these genes leads to reactive oxygen species (ROS) accumulation during the early stage of infection, with the subsequent limitation of the pathogen’s growth. In the presence of a compatible interaction of SnTox3*–Snn3-B1*, ABA signaling is suppressed. On the contrary, in the presence of a compatible interaction of *SnToxA–Tsn1*, ABA signaling is activated, but the activity of the early post-invasive defense genes *TaERD15* and *TaABI5* is inhibited, and the expression of the NAC (NAM, ATAF1/2, and CUC2) transcription factor (TF) family genes *TaNAC29* and *TaNAC21/22* is induced. The TF genes *TaNAC29* and *TaNAC21/22* in the presence of SnToxA induce the development of the susceptibility of wheat plants to *S. nodorum* SnB, associated with a decrease in the oxidative burst during the early stage of infection. Thus, our study provides new data on the role of the NEs SnTox3 and SnToxA in manipulating ABA signaling in the development of the susceptibility of wheat to *S. nodorum*. Deepening our knowledge in this area will be instrumental for developing new strategies for breeding programs and will contribute to the development of environmentally friendly sustainable agriculture.

## 1. Introduction

Plants are constantly threatened by a variety of pathogenic organisms, causing diseases and crop losses. However, plants have evolved immune systems and various defense mechanisms and, in order to cause disease, pathogens must avoid or suppress plant defenses. This “war” occurs at several levels as described in the widely cited “zig-zag” model of immunity. The activation of pattern-triggered immunity (PTI), which restricts pathogen growth, occurs when conserved pathogen-associated molecular patterns (PAMPs) are recognized by plant cell-surface pattern recognition receptors (PRRs) [1,2]. Pathogens avoid or suppress PTI through secreted effector molecules, which results in effector-triggered susceptibility (ETS). Plants, in turn, have evolved intracellular nucleotide-binding leucine-rich repeat receptors (NLRs) to detect effectors, which are often encoded by *Resistance* (R) genes, and activate effector-triggered immunity (ETI) upon effector perception [1,2]. ETI corresponds to a specific gene-for-gene response during biotrophic pathogens attack [1]. The effectors of necrotrophic pathogens suppress PTI and use the host’s ETI pathway to develop susceptibility, resulting in NE-triggered susceptibility (NETS), and this corresponds to the inverse gene-for-gene interaction [3,4].

Over the past 20 years, there have been many reports on the interaction of PRR- mediated immunity (or PTI) and NLR-mediated immunity (or ETI), in other words, the interaction of non-specific and specific immune responses [2]. Thus, ETI can restore or protect the PTI pathway from the action of pathogen effectors. The activation of PRRs can induce the expression of NLRs and NLR-signaling components. The induction of NLRs enhances PAMP-induced responses, such as reactive oxygen species (ROS) production, callose deposition, and defense-related gene expression [2,5,6]. However, the mechanisms of these relationships are not fully understood [2,7]. It has recently been shown that NEs can hijack the non-specific host defense signaling pathways and manipulate them for pathogen growth and development [8,9,10]. However, this question remains open for further study.

PRR- and NLR-mediated immunity is tightly regulated by many mechanisms at multiple levels, as the excessive activation of PRRs and NLRs leads to autoimmunity and growth inhibition. The regulation of immune responses is carried out by plant proteins, pathogen effectors, microRNAs and phytohormones at the level of transcription, and translation and posttranslational modifications (PTMs) [2,11]. However, the fine regulation of PRR- and NLR-signaling components remains to be investigated.

A number of phytohormones are involved in the response of various signaling systems. An infection with a pathogen causes a fairly rapid and intense accumulation of one or more phytohormones, which induce the synthesis of various protective compounds, which leads to the development of plant resistance. Salicylic acid (SA), jasmonic acid (JA), and ethylene (ET) are classic phytohormones involved in the processes of the formation of immunity to pathogens and actively interact with the phytohormones regulating plant growth and development, such as cytokinins (CKs), auxins, abscisic acid (ABA), and others [11,12,13,14]. Until recently, growth and development phytohormones were considered in plant immunity from the perspective of the formation of compatible interactions with pathogens, missing the positive role of growth regulators in the development of immune responses [11,12]. At the same time, in the last decade, much evidence has emerged of the involvement of such phytohormones (CK, auxins, ABA, etc.) in the regulation of plant–microbial interactions, through positive or negative effects on the activity of the components of the signaling pathways of SA, JA, and ET [11,13,14].

ABA is a plant hormone of a terpenoid nature, the main functions of which are the inhibition of plant growth and the regulation of gas exchange, as well as the adaptation of plants to abiotic stresses [15,16,17]. Current knowledge about the effect of ABA on plant immunity is insufficient and contradictory [15,16,18]. The model of ABA action proposed by Ton et al. (2004) suggests a positive role of the phytohormone in the development of defense reactions in the early stages of infection, expressed through ABA-dependent stomatal closure and callose accumulation, and a negative role in the late stages of colonization, associated with the suppression of the SA- and JA-dependent signaling pathways [19]. Numerous studies have shown both a positive and negative role of ABA in plant protection against pathogens [20,21,22,23]. Fungi, such as *Magnaporthe oryzae*, *Ustilago maydis,* and *Botrytis cinerea,* have been shown to be able to synthesize ABA [21,22]. ABA has also been shown to suppress the immune response of tomatoes infected with *B. cinerea*, barley infected with *M. oryzae*, and tobacco infected with *Ralstonia solanacearum* [23]. On the other hand, plants require the ABA signaling pathway for resistance to several necrotrophic pathogens, including *Pythium corrective*, *Plectosphaerella cucumerina*, *Cochliobolus miyabeanus,* and *Alternaria brassicicola* [19,20,22,24]. The dual role of ABA in plant defense apparently depends upon the infection phase and pathogen lifestyle [23]. It seems that ABA may influence the penetration and proliferation of pathogens, as well as the expression of the genes that encode the proteins associated with plant responses to biotic stress [16,18]. However, the precise mechanisms of the ABA-mediated response to biotic stress are still unknown [16,23].

*Stagonospora nodorum*, an ascomycete fungus (teleomorph: *Parastagonospora nodorum*), is the causal agent of wheat Stagonospora nodorum blotch (SNB), a globally distributed and economically important disease [25,26,27]. *S. nodorum* produces NEs, which are major virulence factors. They interact with the products of the dominant host plant’s susceptibility genes (*Snns*) and lead to the development of SNB [25]. The effectors SnToxA, SnTox1, and SnTox3 are quite widespread among strains and isolates, cause necrosis and chlorosis in susceptible wheat genotypes, and their role in the suppression of PTI and the development of NETS is being actively studied [25,26,28,29,30]. SnToxA interacts with *Tsn1*, SnTox1 and SnTox3 interact with *Snn1* and *Snn3*, respectively, resulting in cell death [8,9,10,26,31]. The host genes *Tsn1* and *Snn1* have been cloned and encode a serine/threonine protein kinase, a nucleotide-binding site leucine-rich repeat protein (NB-LRR), and a wall-associated kinase protein (WAK), respectively [10,32].

It was previously shown that the effector SnTox1 can suppress PTI by binding to chitin and by preventing plant chitinases from binding and degrading the fungal cell wall [8]. The effectors SnToxA and SnTox3 influence the SA signaling pathway through direct interaction with PR1, which can lead to the suppression of PTI and increased susceptibility to *S. nodorum* [28]. Previously, we showed that the NE SnTox3 induces the ethylene signaling pathway, regulating ROS production in wheat in the early stage of an *S. nodorum* infection. Our previous results suggest that the activation of the ethylene signaling pathway is aimed at the suppression of SA-dependent defense responses and PTI in wheat susceptible to *S. nodorum* isolates producing SnTox3 [29]. Our studies also showed that SnTox1 hijacks the SA pathway to suppress catalase activity, increase the hydrogen peroxide content, and induce necrosis formation; it simultaneously suppresses the JA and ethylene hormonal pathways by SA [30]. Nevertheless, the role of hormonal signaling pathways in regulating the mechanisms of action of *S. nodorum* NEs, leading to cell death, remains unclear.

We hypothesize that the compatible interaction of SnToxA–*Tsn1* can play a negative role in the effect of ABA on plant defense responses or, in other words, the *S. nodorum* SnB isolate with the help of the NE SnToxA can manipulate the ABA signaling pathway for its growth and development. Previously, we showed that the presence of a compatible interaction of SnToxA–*Tsn1* causes an increase in catalase (CAT) activity, which leads to a decrease in ROS production and the development of the susceptibility of wheat plants to the SnB isolate [33].

In this regard, the goal of our work was to identify the role of ABA and the ABA signaling pathway in the development of resistance/susceptibility, associated with the regulation of the redox status of plants, to the *S. nodorum* SnB isolate, expressing the *SnToxA* and *SnTox3* genes, in three wheat genotypes (*Triticum aestivum* L.) with different sensitivities to NEs. For this, the generation of hydrogen peroxide (H_2_O_2_), the activity of the CAT enzyme, and the endogenous content of the phytohormone ABA were studied in three different cultivars of bread wheat pretreated with the phytohormone and inoculated with the *S. nodorum* SnB isolate. We also analyzed the expression of the NEs *SnToxA* and *SnTox3* genes in the three wheat genotypes pretreated with the phytohormone ABA and inoculated with the *S. nodorum* SnB isolate. Additionally, to study the effect of the NEs SnToxA and SnTox3 on the ABA signaling pathway, we analyzed the transcriptional activity of eight genes involved in the biosynthesis and signaling pathway of ABA in the leaves of three different wheat cultivars pretreated with the phytohormone and inoculated with the *S. nodorum* SnB isolate.

Thus, our results show that the role of ABA in the wheat–*S. nodorum* interaction depends on both the plant genotype and the production of NEs by the pathogen isolate, which reveals the complex regulation of the interactions between the NEs in the pathogen *S. nodorum*. And, our study provides new data on the role of the NEs SnTox3 and SnToxA in manipulating ABA signaling for the development of the susceptibility of wheat to *S. nodorum*.

## 2. Results

### 2.1. The Role of SnTox3, SnToxA, and Phytohormone ABA in the Development of Defense Reactions in the Three Wheat Genotypes

In this work, the defense reactions of three bread wheat (*Triticum aestivum* L.) genotypes were studied during ABA treatment and infection with the isolate of *S. nodorum* SnB expressing two NE genes, *SnToxA* and *SnTox3*. The cv. Omskaya 35 (Om35) is resistant to *S. nodorum* due to its insensitivity to both SnToxA and SnTox3, as it has recessive alleles for both susceptibility genes (*tsn1*/*snn3*) [34]. The cultivar Kazakhstanskaya 10 (Kaz10) is sensitive to the NE SnTox3 (*tsn1*/*Snn3*), meaning that the cv. Kaz10 has one specific compatible interaction of SnTox3–*Snn3* [34]. The cv. Zhnitsa (Zhn) is susceptible to both NEs SnToxA and SnTox3 (*Tsn1*/*Snn3*), meaning the susceptibility of Zhnitsa to *S. nodorum* is determined by two compatible, specific gene-for-gene interactions (SnToxA–*Tsn1* and SnTox3–*Snn3*) [34].

**Figure 1:**
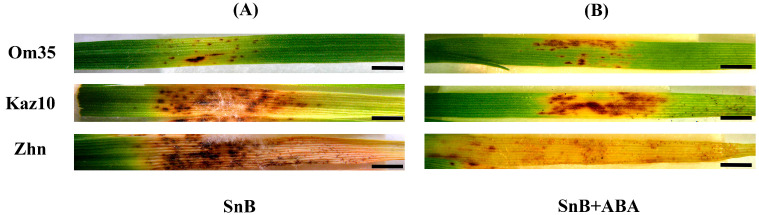
The effect of ABA treatment on symptom development of leaves of the three wheat genotypes infected with *S. nodorum* SnB. (**A**) Wheat plants not treated with ABA and infected with the SnB isolate (SnB); (**B**) wheat plants treated with 2 µm ABA and infected with the SnB isolate (SnB + ABA). Wheat cultivars designations: Omskaya 35 (Om35), Kazahstanskaya 10 (Kaz10), and Zhnitsa (Zhn). The photographs show the results of a typical variant from a series of experiments (*n* = 30). The development of symptoms was recorded 8 days after infection. Bars = 15 mm.

The analysis of the phenotypic manifestation of SNB showed that the *S. nodorum* SnB isolate affected only 8% of the leaf area in the cv. Om35 (Figure 1, Table 1). For cv. Kaz10, the leaf-damaged areas occupied more than 50% of the total leaf area (61%), and for cv. Zhnitsa, the lesion zones occupied 81% of the leaf area (Figure 1, Table 1).

Thus, the plants of cv. Om35 showed resistance to the *S. nodorum* SnB isolate, and the plants of cultivars Kaz10 and Zhnitsa showed susceptibility to this isolate (Figure 1, Table 1). However, cv. Kaz10 was more resistant to the *S. nodorum* SnB isolate than cv. Zhnitsa, which had a much larger area of lesions than cv. Kaz10 (Figure 1, Table 1).

The effect of ABA on the development of defense reactions in the plants depended on the genotype of the plant. In cv. Zhnitsa (*Tsn1/Snn3*), the ABA treatment led to an increase in the damaged zones to 97% (Figure 1, Table 1). In cv. Om35 (*tsn1/snn3*), the ABA treatment did not affect the affected areas, it remained at the same minimum of 9% (Figure 1, Table 1). In cv. Kaz10 (*tsn1/Snn3*), the ABA treatment led to a decrease in the lesion zones, to 29%.

**Figure 2:**
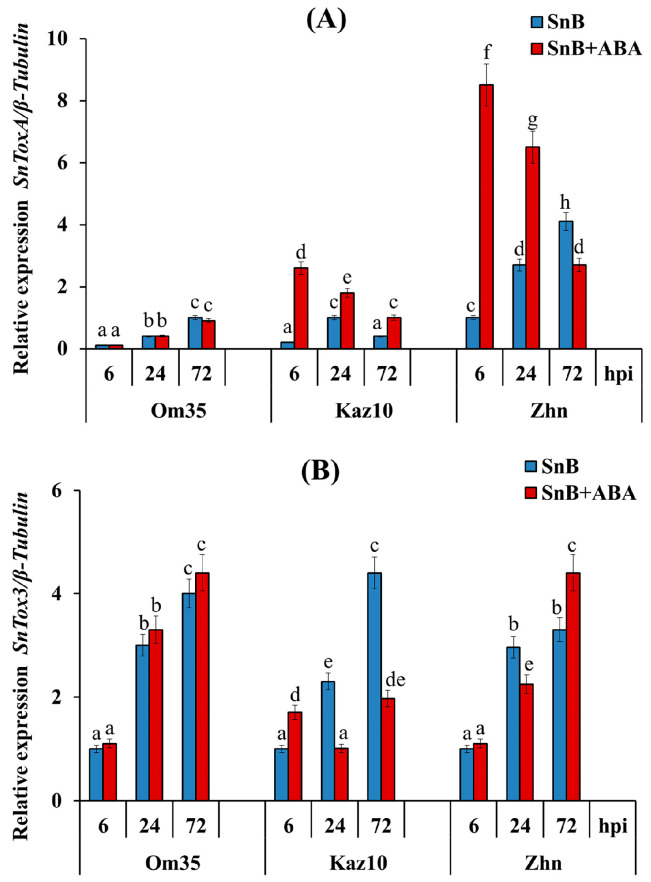
Comparative analysis of the *SnTox* genes’ expression in three bread wheat cultivars during treatment with ABA and infection with SnB isolate. (**A**) The transcript level of the *SnToxA* gene normalized to *β-tubulin*; (**B**) the transcript level of the *SnTox3* gene normalized to *β-tubulin*. Designations: SnB—wheat plants untreated with phytohormone and infected with *S. nodorum* isolate SnB; SnB + ABA—wheat plants treated with abscisic acid (ABA) and infected with *S. nodorum* isolate SnB. Wheat cultivars designations: Omskaya 35 (Om35), Kazahstanskaya 10 (Kaz10), and Zhnitsa (Zhn). Average gene expression is calculated from nine biological samples of five technical replicates (*n* = 9). Values are expressed as mean SE (ANOVA with Duncan’s test; comparisons between genotypes are presented; significant differences are marked with different letters at *p* ≤ 0.05).

In this work, the relative expression of two genes, the NEs *SnToxA* and *SnTox3*, to the fungal *β-tubulin* gene was examined in three infected wheat cultivars under an ABA treatment using a real-time quantitative PCR (Figure 2). The two cultivars Om35 and Kaz10, insensitive to the NE SnToxA, showed low transcript levels of the *SnToxA* gene relative to the fungal *β-tubulin* gene at 6, 24, and 72 h post infection (hpi) (Figure 2A).

In the susceptible cv. Zhnitsa, the expression of the *SnToxA* gene increased gradually, reached its maximum at 72 hpi, and exceeded the expression of the *β-tubulin* gene by four times (Figure 2A). The ABA treatment of plants did not affect the *SnToxA* gene’s mRNA abundance in the resistant cv. Om35 compared to the infected, untreated plants (Figure 2A). However, the ABA treatment of plants increased the *SnToxA* gene expression in the infected cultivars Kaz10 and Zhnitsa, with maximum transcript levels after 6 hpi, exceeding the *β-tubulin* gene expression by 2.6 and 8.5 times, respectively (Figure 2A). Then, the *SnToxA* gene expression gradually decreased (Figure 2A). Thus, the highest transcript levels of the *SnToxA* gene were detected during the infection of the cv. Zhnitsa carrying the *Tsn1* susceptibility gene to the NE SnToxA in its genome.

All three cultivars, Om35, Kaz10, and Zhnitsa, regardless of genotype, showed the same pattern of *SnTox3* gene expression during infection with the SnB isolate (Figure 2). The maximum transcript level of the *SnTox3* gene was detected at 72 hpi in all the cultivars, and exceeded the expression of the *β-tubulin* gene by approximately four times (Figure 2B).

The treatment of the plants with ABA did not affect the mRNA abundance of the *SnTox3* gene in the absence of a compatible interaction of SnTox3-*Snn3-B1* in the resistant cv. Om35 compared to the infected, untreated plants (Figure 2,B). However, the ABA treatment of the plants differently regulated the expression of the *SnTox3* gene in the cv. Kaz10 (SnTox3–*Snn3-B1*) and in the cv. Zhnitsa (SnToxA–*Tsn1* and SnTox3–*Snn3-B1*) in the presence of compatible interactions. The ABA treatment of the Kaz10 plants led to an increase in the mRNA abundance of the *SnTox3* gene at 6 hpi and to a decrease in the transcript level of this gene at 24 and 72 hpi compared to the infected, untreated plants (Figure 2B). In contrast, the ABA treatment of the cv. Zhnitsa plants had no effect on the expression of the *SnTox3* gene at 6 and 24 hpi and increased the mRNA abundance of this gene at 72 hpi compared to the infected, untreated plants (Figure 2B).

### 2.2. The Role of SnTox3, SnToxA, and Phytohormone ABA in the Regulation of the Redox Status of the Three Wheat Genotypes

**Figure 3:**
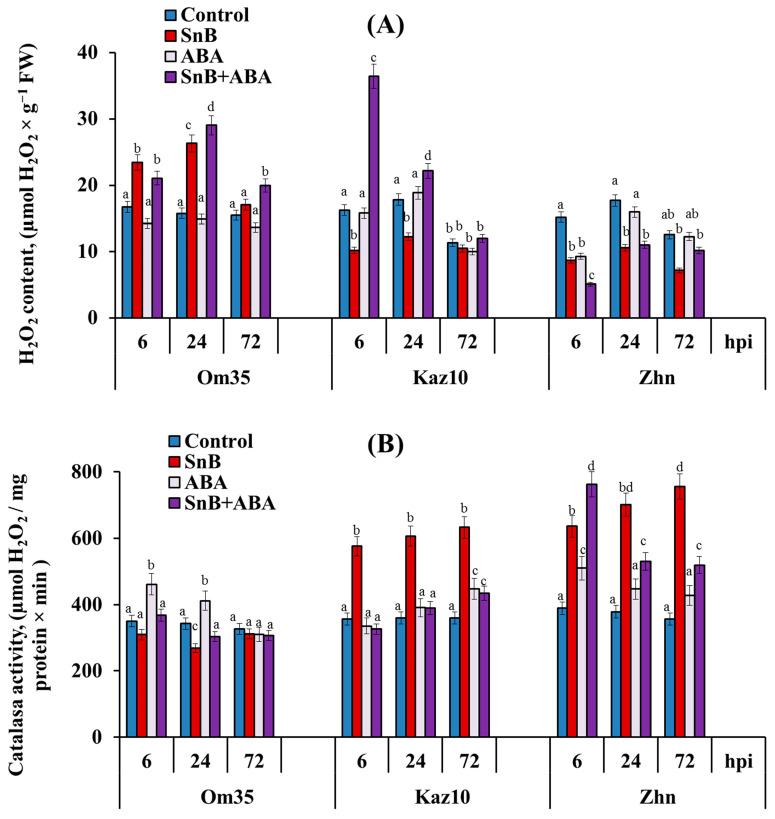
Effect of ABA treatment on changes in H_2_O_2_ content (**A**) and activity of catalase (**B**) enzyme in three cultivars of bread wheat at 6, 24, and 72 hpi with the isolate *S. nodorum* SnB. Designations: Control—untreated and uninfected wheat plants; SnB—wheat plants untreated with phytohormone and infected with *S. nodorum* isolate SnB; ABA—wheat plants treated with abscisic acid (ABA) and uninfected wheat plants; SnB + ABA—wheat plants treated with abscisic acid and infected with *S. nodorum* isolate SnB; FW—fresh weight. Wheat cultivars designations: Omskaya 35 (Om35), Kazahstanskaya 10 (Kaz10), and Zhnitsa (Zhn). Figures present means ± SE (*n* = 9) (ANOVA with Duncan’s test; comparisons with the control for each genotype are presented; significant differences are marked with different letters at *p* ≤ 0.05).

It was previously established that the resistance of *T. aestivum* to the *S. nodorum* SnB isolate is determined by the intensive generation of ROS, mainly H_2_O_2_, due to the lack of an increase in CAT activity during the initial stage of infection [33]. The results of this work show that in the resistant cv. Om35, the H_2_O_2_ content was already increased at 6 and 24 hpi. (Figure 3A). The ABA treatment of the Om35 plants did not affect the H_2_O_2_ content during infection compared to the infected, untreated plants (Figure 3A). In the cultivars Kaz10 and Zhnitsa, which have compatible interactions, a different reaction was observed. Infection with the isolate SnB led to a decrease in the H_2_O_2_ content in both cultivars at 6 and 24 hpi (Figure 3A). In the cv. Zhnitsa, the H_2_O_2_ content also decreased at 72 hpi (Figure 3A). However, the treatment of the plants with ABA had different effects on ROS generation in these cultivars. In the cultivar Kaz10 (*tsn1/Snn3*), the ABA treatment resulted in more than a twofold accumulation of H_2_O_2_ at 6 hpi and a small accumulation of H_2_O_2_ at 24 hpi (Figure 3A). In the cv. Zhnitsa (*Tsn1/Snn3*), the ABA treatment led to a decrease in the H_2_O_2_ content at 6 hpi, but did not affect this indicator at 24 or 72 hpi compared to the infected, untreated plants (Figure 3A). The effect of ABA on H_2_O_2_ generation in the infected plants depended on the plant genotype.

The analysis of the CAT activity showed that in the resistant cv. Om35, CAT activity did not increase at 6, 24, or 72 hpi (Figure 3B). In the cultivars Kaz10 and Zhnitsa with compatible interactions, infection with the *S. nodorum* SnB isolate led to an increase in CAT activity at 6, 24, and 72 hpi (Figure 3B). The treatment of plants with ABA slightly increased CAT activity in all the cultivars in the absence of infection (Figure 3B). However, in the presence of the *S. nodorum* SnB isolate, ABA had different effects on the enzyme activity of the different wheat genotypes. Thus, the ABA treatment of the Om35 plants did not affect CAT activity during infection compared to the infected, untreated plants (Figure 3B). In the susceptible cv. Kaz10, treatment with ABA reduced the activity of CAT by almost two times compared to the infected, untreated plants at 6, 24, and 72 hpi (Figure 3B), which led to an increase in H_2_O_2_ content and the development of defense reactions (Figure 1, Table 1).

In the susceptible cv. Zhnitsa, the ABA treatment increased CAT activity at 6 hpi, but decreased enzyme activity at 24 and 72 hpi compared to the infected, untreated plants (Figure 3B). The increase in enzyme activity at 6 hpi in the ABA-treated plants was much greater than without the ABA treatment (Figure 3B). This could lead to a decrease in H_2_O_2_ content and the development of large lesions (Figure 1, Table 1).

### 2.3. The Role of SnTox3 and SnToxA in the Regulation of the Endogenous ABA Content in the Three Wheat Genotypes

**Figure 4:**
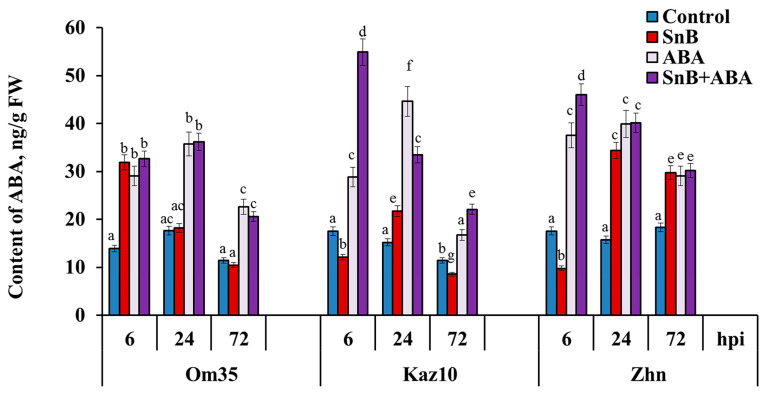
The endogenous ABA content in the three wheat genotypes infected with the *S. nodorum* SnB isolate and treated with 2 μM ABA. Symbols are the same as in Figure 3. Figures present means ± SE (*n* = 9) (ANOVA with Duncan’s test; comparisons with the control for each genotype are presented; significant differences are marked with different letters at *p* ≤ 0.05).

In this work, the endogenous ABA content was analyzed using an ELISA for the three wheat genotypes infected with the *S. nodorum* SnB isolate and treated with 2 μM ABA. The ABA content increased by 2.3 times at 6 hpi compared to the control only in the resistant cv. Om35 (Figure 4). In the susceptible cvs. Kaz10 (SnTox3–*Snn3*) and Zhnitsa (SnToxA–*Tsn1* and SnTox3–*Snn3*), the ABA content in the infected leaves at this time decreased by 1.5 and 1.8 times, respectively, compared to the control (Figure 4).

Then, the ABA content in the resistant cv. Om35 did not change at 24 or 72 hpi compared to the control (Figure 4). On the contrary, during the same infection period (24 and 72 hpi), the susceptible cv. Zhnitsa showed a 2.2- and 1.7-fold increase in the ABA content, respectively, compared to the control (Figure 4). In the susceptible cv. Kaz10, an ABA accumulation of 1.4 times compared to the control was detected only at 24 hpi, and the ABA content in this cultivar decreased at 72 hpi (Figure 4).

The treatment of the plants with ABA led to an increase in the endogenous level of the hormone in the leaves of all three cultivars under control conditions and during infection with the *S. nodorum* SnB isolate, but the dynamics of hormone accumulation had a diverse pattern in the different genotypes (Figure 4). In the resistant genotype Om35, the ABA treatment did not affect the hormone content at 6 hpi and increased its content at 24 and 72 hpi compared to the infected, untreated plants (Figure 4). In the susceptible genotype Kaz10 (SnTox3–*Snn3*), the ABA treatment increased the hormone content by 4.6, 2.2, and 2.6 times at 6, 24, and 72 hpi, respectively, compared to the infected, untreated plants (Figure 4). In the susceptible genotype Zhnitsa (SnToxA–*Tsn1* and SnTox3–*Snn3*), the ABA treatment increased the hormone content by 4.7 times at 6 hpi and did not affect its content at 24 or 72 hpi compared to the infected, untreated plants (Figure 4).

### 2.4. The Role of SnTox3 and SnToxA in Manipulating the ABA Signaling Pathway and the Role of the Hormone ABA in the Development of the Resistance/Susceptibility of Wheat to the S. nodorum SnB Isolate

To study the role of ABA in the development of plant resistance or susceptibility to the *S. nodorum* SnB isolate, the transcriptional activity of eight genes in the ABA biosynthesis and signaling pathways was studied. The expression of the genes involved in two stages of ABA biosynthesis was analyzed. The expression of the gene encoding the enzyme zeaxanthin epoxidase (*TaZEP*), which converts zeaxanthin into violaxanthin through an epoxidation reaction, was studied; this step of ABA biosynthesis occurs in plastids [15]. The expression of the gene encoding the enzyme 9-cis-epoxycarotenoid dioxygenase (*TaNCED1*), which catalyzes the oxidative cleavage of 9-cis-violaxanthin and/or 9-cis-neoxantin to C15 cis-xanthoxin and C25 metabolite, was studied [15,16]. This is the last reaction of the ABA biosynthesis pathway, which occurs in chloroplasts and is the rate-limiting step of ABA biosynthesis. The signaling module PYR/PYL/RCAR (pyrabactin resistance1/PYR1-like/regulatory component of ABA receptors), -PP2C (protein phosphatase 2C), -SnRK2 (sucrose non-fermenting 1-related protein kinase 2) is generally agreed to be a key player in the recognition of ABA and further signal transduction [15,16]. We studied the expression of the gene encoding the regulatory component of ABA (*TaRCAR*) and the *TaSnRK2.10* gene, which encodes a positive regulator of ABA signaling. SnRK2 activates by phosphorylating a group of downstream transcription factors (TFs), including ABA-insensitive 5 (ABI5) and early responsive to dehydration 15 (ERD15) [16,35]. We also studied the expression of the ABA signaling TF genes *TaERD15* and *TaABI5*. The proline oxidase ERD15 stimulates post-invasive defense but suppresses ABA-dependent abiotic stress tolerance, thereby acting as a branching point in the crosstalk between biotic and abiotic stress [36]. ABI5 is a basic leucine zipper TF that plays a key role in the positive regulation of early post-invasive defense [37]. Moreover, ABI5 also acts as an ABA and another phytohormone signaling integrator [38]. The expression of two genes (*TaNAC21/22*, *TaNAC29*) from the largest plant-specific TF superfamily, NAM, ATAF1/2, and CUC2 (NAC) TFs, which play important roles in various physiological processes, including stress responses and are induced by ABA, was also investigated [39,40,41].

**Figure 5:**
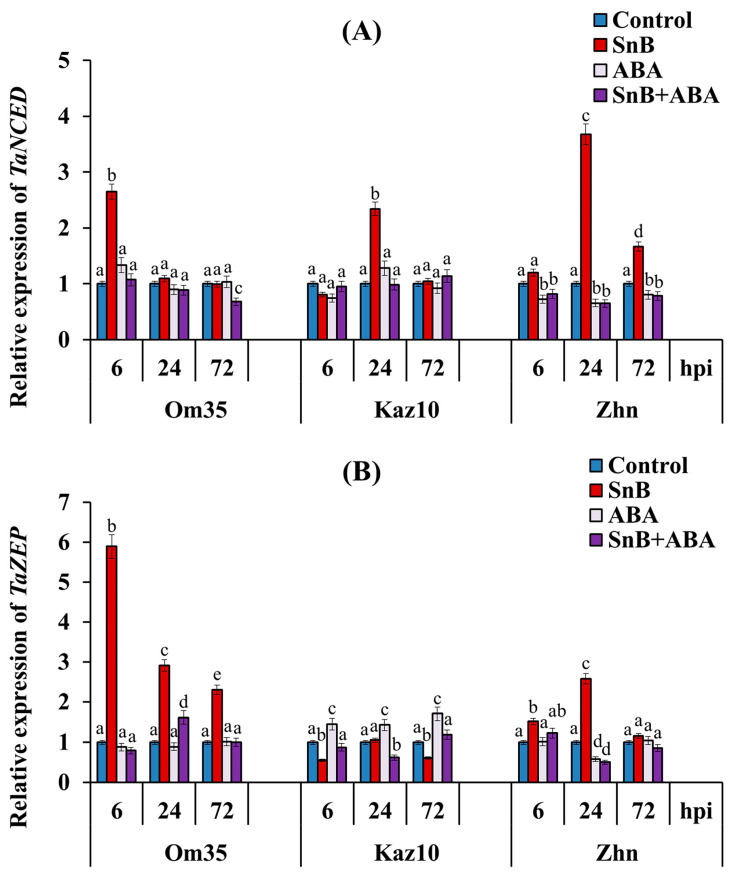
Effect of ABA treatment on expression of two genes of ABA biosynthesis—*TaNCED1* (**A**) and *TaZEP* (**B**) in three bread wheat cultivars 6, 24, and 72 hpi with isolate *S. nodorum* SnB. Symbols are same as in Figure 3. Average gene expression is calculated from nine biological samples of five technical replicates (*n* = 9). Values are expressed as mean ± SE (ANOVA with Duncan’s test; comparisons with control for each genotype are presented; significant differences are marked with different letters at *p* ≤ 0.05).

The results of this work show that infection with the SnB isolate induced the transcript accumulation of the *TaNCED1* gene in all three cultivars by 2–3.6 times compared to the control (Figure 5A). However, in the resistant cv. Om35, the transcript level of the *TaNCED1* gene increased only at 6 hpi; in the cv. Kaz10, only at 24 hpi; and in the cv. Zhnitsa, at 24 and 72 hpi (Figure 5A). The mRNA content of the *TaZEP* gene increased in the cv. Om35 by 6, 3, and 2.3 times, respectively, at 6, 24, and 72 hpi (Figure 5B). In the cv. Zhnitsa, an increase of 1.5 and 2.6 times in the transcript level of this gene was detected at 6 and 24 hpi and in the cv. Kaz10, the transcript level of the *TaZEP* gene decreased by almost 2 times at 6 and 72 hpi compared to the control (Figure 5B). The treatment of the plants with ABA in most cases did not affect or decrease the transcript level of the *TaNCED1* and *TaZEP* genes in all three cultivars under control conditions and during infection with the *S. nodorum* SnB isolate, compared to the control (Figure 5). Most likely this was due to the high level of ABA in the plants treated with the hormone according to the feedback principle, which was confirmed by the ELISA results (Figure 4).

**Figure 6:**
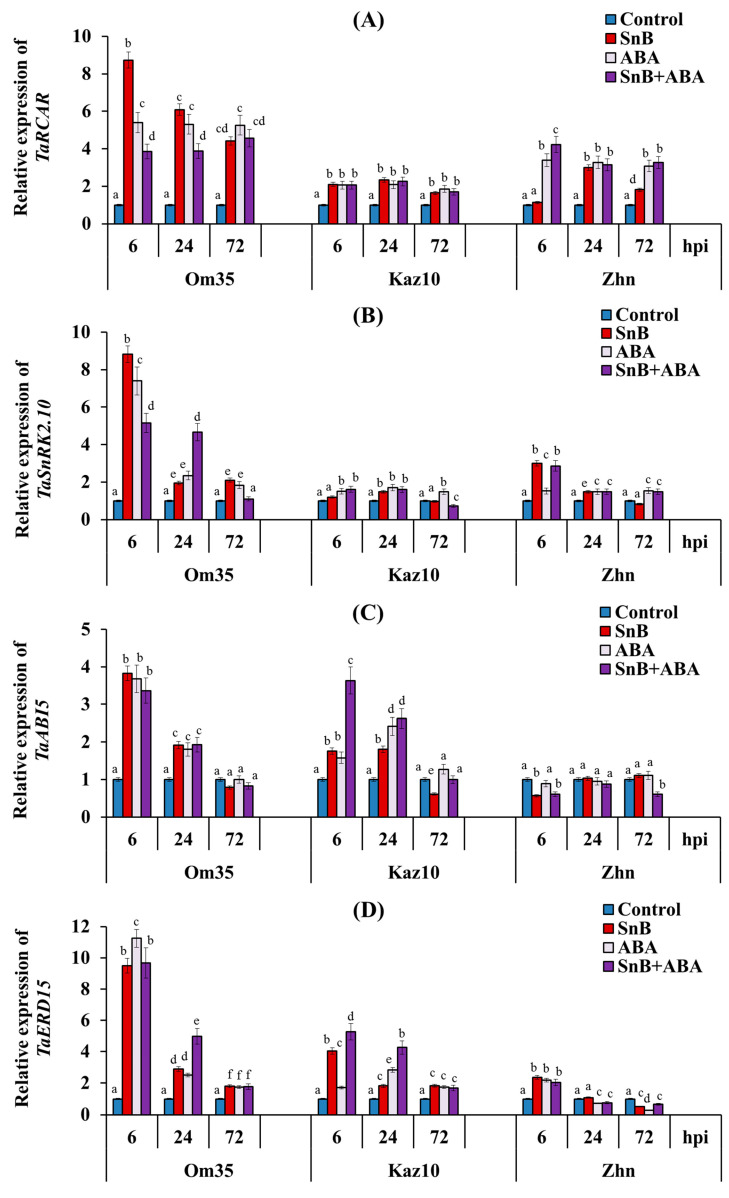
Effect of ABA treatment on the expression of four genes of ABA signaling— *TaRCAR* (**A**), *TaSnRK2.10* (**B**), *TaABI5* (**C**), and *TaERD15* (**D**)— in three bread wheat cultivars at 6, 24, and 72 hpi with the isolate *S. nodorum* SnB. The symbols are the same as in Figure 3. Average gene expression is calculated from nine biological samples from five technical replicates (*n* = 9). Values are expressed as mean ± SE (ANOVA with Duncan’s test; comparisons with the control for each genotype are presented; significant differences are marked with different letters at *p* ≤ 0.05).

The results of this work show that the transcript level of the *TaRCAR* gene increased in all three cultivars during infection with the *S. nodorum* SnB isolate, compared to the control (Figure 6A). However, in the resistant cv. Om35, the mRNA content of this gene increased extremely by 8.7 and 6 times at 6 and 24 hpi, respectively, and in the susceptible cultivars Kaz10 and Zhnitsa, a 2- to 3-fold increase in the expression of this gene was found compared to the control (Figure 6A). It is worth noting that in the susceptible cv. Zhnitsa, the transcript level of the *TaRCAR* gene did not increase at 6 hpi compared to the control (Figure 6A).

The treatment of the plants with ABA led to an increase in the transcript level of the *TaRCAR* gene in all three cultivars under control conditions and during infection with the *S. nodorum* SnB isolate, compared to the control (Figure 6A). However, in comparison with the infected untreated plants, the expression pattern of the *TaRCAR* gene differed in the diverse cultivars treated with ABA (Figure 6A). In the resistant genotype Om35, the ABA treatment led to a decrease in the mRNA content of the *TaRCAR* gene at 6 and 24 hpi compared to the infected, untreated plants (Figure 6A).

In the susceptible genotype Zhnitsa, the ABA treatment increased the transcript level of the *TaRCAR* gene by 4 and 1.8 times at 6 and 72 hpi, respectively, compared to the infected, untreated plants (Figure 6A). In all the other variants, the ABA treatment did not affect the expression of this gene compared to the infected, untreated plants (Figure 6A). The transcript level of the *TaSnRK2.10* gene in the resistant cv. Om35 increased at 6, 24, and 72 hpi compared to the control; an extreme 9-fold increase in the mRNA content of this gene was detected at 6 hpi compared to the control (Figure 6B). In the susceptible cv. Zhnitsa, an increase in the transcript level of the *TaSnRK2.10* gene of 3 and 1.5 times was also detected at 6 and 24 hpi, respectively, compared to the control (Figure 6B). In the susceptible cv. Kaz10, the expression of the *TaSnRK2.10* gene increased only at 24 hpi and by only 1.5 times compared to the control (Figure 6B). The treatment of the plants with ABA led to an increase in the transcript level of the *TaSnRK2.10* gene in all three cultivars under control conditions and during infection with the *S. nodorum* SnB isolate, compared to the control (Figure 6B). However, in comparison with the infected untreated plants, the ABA treatment led to a decrease in the mRNA content of this gene in the cv. Om35 at 6 and 72 hpi and in the cv. Kaz10 at 72 hpi, and led to an increase in the transcript level of the *TaSnRK2.10* gene in the cv. Kaz10 at 6 hpi and in the cv. Zhnitsa at 72 hpi (Figure 6B).

The expression pattern of the *TaABI5* and *TaERD15* genes was similar in each cultivar (Figure 6C,D). In the resistant cv. Om35, the mRNA content of the *TaABI5* and *TaERD15* genes increased at 6 and 24 hpi by 4 and 2 times and 9.5 and 3 times, respectively, compared to the control (Figure 6C,D). Additionally, in the cv. Om35, a 2-fold increase in the transcript level of the *TaERD15* gene was detected at 72 hpi compared to the control (Figure 6D). The treatment of plants with ABA did not affect the expression of the *TaABI5* and *TaERD15* genes in the cv. Om35 compared to the infected, untreated plants (Figure 6C,D).

In the susceptible cv. Kaz10, the mRNA content of the *TaABI5* and *TaERD15* genes increased by 2–4 times at 6 and 24 hpi compared to the control (Figure 6C,D). Additionally, in the cv. Kaz10, an increase in the transcript level of the *TaERD15* gene of 1.8 times was detected at 72 hpi compared to the control (Figure 6D). The treatment of the plants with ABA led to an increase in the transcript level of these genes by 1.5–2-fold at 6 and 24 hpi compared to the infected, untreated plants (Figure 6C,D). The treatment of plants with ABA did not affect the expression of the *TaABI5* and *TaERD15* genes in the cv. Kaz10 at 72 hpi compared to the control (Figure 6D). In the susceptible cv. Zhnitsa, a 2-fold decrease in the transcript level of the *TaABI5* gene was found at 6 hpi, and the expression of this gene did not change further compared to the control (Figure 6C). In contrast, the mRNA content of the *TaERD15* gene in the cv. Zhnitsa increased 2.4-fold at 6 hpi and decreased 2-fold at 72 hpi compared to the control (Figure 6D). The treatment of the plants with ABA led to a 2-fold decrease in the transcript level of the *TaABI5* gene at 72 hpi and did not affect the expression of the *TaERD15* gene in the cv. Zhnitsa compared to the infected, untreated plants (Figure 6C,D).

**Figure 7:**
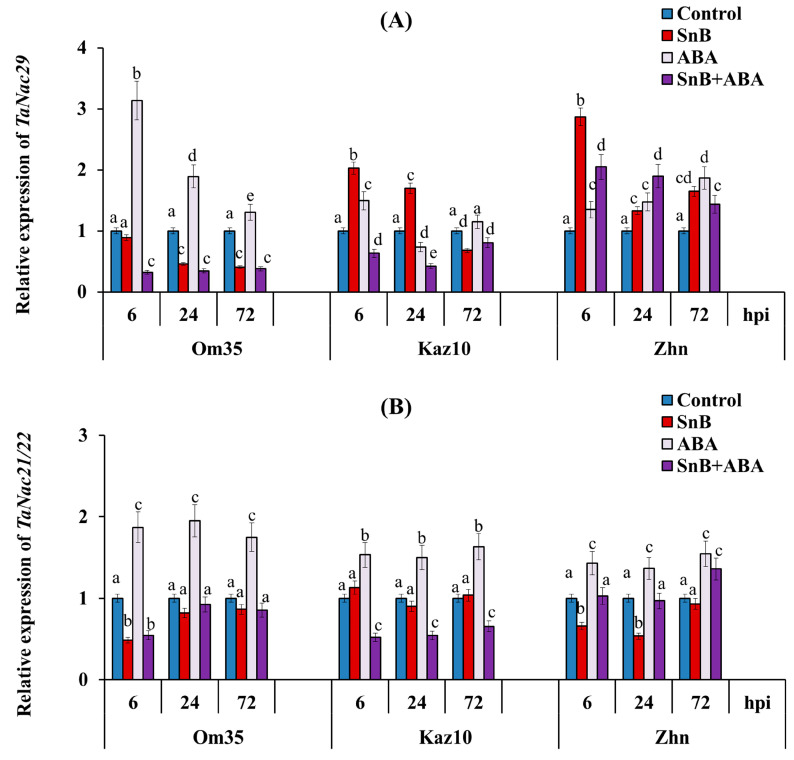
Effect of ABA treatment on expression of two ABA-inducible TF genes—*TaNAC29* (**A**) and *TaNAC21/22* (**B**)— in three bread wheat cultivars at 6, 24, and 72 hpi with isolate *S. nodorum* SnB. Symbols are same as in Figure 3. Average gene expression is calculated from nine biological samples from five technical replicates (*n* = 9). Values are expressed as mean ± SE (ANOVA with Duncan’s test; comparisons with control for each genotype are presented; significant differences are marked with different letters at *p* ≤ 0.05).

The infection of the plants with the *S. nodorum* SnB isolate induced an increase in the transcript level of the *TaNAC29* gene only in the susceptible genotypes Kaz10 and Zhnitsa, by 1.3–3 times, compared to the control (Figure 7A). The strongest increase in the mRNA content of the *TaNAC29* gene of three times was found in the cv. Zhnitsa at 6 hpi compared to the control (Figure 7A). In the resistant genotype Om35, the transcript level of the *TaNAC29* gene decreased at 24 and 72 hpi compared to the control (Figure 7A). The treatment of the plants with ABA under control conditions induced the expression of the*TaNAC29* gene in all three cultivars compared to the untreated control plants (Figure 7A). However, the ABA treatment did not affect or led to a decrease in the expression of the *TaNAC29* gene in the cultivars Om35 and Kaz10 compared to the infected, untreated plants (Figure 7A). The strongest decrease in the transcript level of the *TaNAC29* gene of 4.1 times was found in the cv. Kaz10 treated with ABA at 24 hpi compared to the infected, untreated plants (Figure 7A). In the susceptible cv. Zhnitsa, the ABA treatment led to an increase in the expression of the *TaNAC29* gene of 1.5–2 times compared to the control, but decreased the level of the transcripts of this gene at 6 and 72 hpi, and increased only at 24 hpi by 1.5 times compared to the infected, untreated plants (Figure 7A).

The analysis of the expression of the *TaNAC21/22* gene showed a decrease in the transcript level of the gene during infection with the *S. nodorum* SnB isolate in the cv. Om35 at 6 hpi and the cv. Zhnitsa at 6 and 24 hpi compared to the control (Figure 7B). In all other variants the expression of this gene did not change compared to the control (Figure 7B). The treatment of plants with ABA under control conditions induced the expression of the *TaNAC21/22* gene in all three cultivars compared to the untreated control plants (Figure 7B). The treatment of plants with ABA during infection with the *S. nodorum* SnB isolate did not affect the expression of the *TaNAC21/22* gene in the cv. Om35, decreased the transcript level of this gene by 1.5 times in the cv. Kaz10, and increased the mRNA content of the TF gene by 1.5–2 times in the cv. Zhnitsa compared to the infected, untreated plants (Figure 7B).

## 3. Discussion

ABA is not only important for plant responses to abiotic stress, but also plays a key and multifaceted role in plant immunity [18]. In the model of the ABA action in the defense response of plants against pathogens proposed by Ton et al. (2004, 2009) and expanded by Cao et al. (2011), the positive or negative role of ABA depends on the phase of the defense response [19,37,42]. Phase I is called pre-invasive penetration defense (or pre-invasive PTI), corresponds to PTI, and continues from one to several hours and consists of limiting the penetration of a pathogen into the host tissues [19,37,42]. In Phase I, ABA stimulates resistance against fungi by mediating the stomatal closure [37]. Phase II is called early post-invasive defense (or post-invasive PTI), and corresponds to PTI, and is characterized by a rapid deposition of callose and generation of ROS. In Phase II, the role of ABA is controversial and varies depending on different plant–pathogen interactions [19,37,42]. Phase III is called late defense (or ABA and ETI), corresponds to ETI, and is characterized by an oxidative burst and the expression of defense-related genes. ABA in this phase suppresses resistance and increases susceptibility [37].

We have previously shown that the fungus *S. nodorum* actively penetrates through the stomata into leaf tissue by 6 h and successfully replicates in the intercellular spaces of the epidermis and mesophyll of the leaves by 24 h after infection, which corresponds to Phase II [29]. Phase III in an *S. nodorum* infection of wheat can be observed at 72 hpi, when the first symptoms of oxidative burst or necrosis appear on the leaves [29].

The resistant cv. Om35 and the susceptible cultivars Kaz10 (SnTox3–*Snn3-B1*) and Zhnitsa (SnToxA–*Tsn1* and SnTox3–*Snn3-B1*) differed in the time and degree of endogenous ABA accumulation in Phase II (6 and 24 hpi) and in Phase III (72 hpi) (Figure 4). In the absence of a compatible interaction, the ABA content in the resistant cv. Om35 increased by 6 hpi and had not changed by 24 hpi (Figure 4). At the same time, the accumulation of H_2_O_2_ and no increase in CAT activity were detected (Figure 3), which triggered a defense response and led to the formation of minimal lesions (Figure 1, Table 1). In the presence of compatible interactions, the ABA levels in the susceptible cultivars (Kaz10 and Zhnitsa) increased by 24 hpi, and in the presence of two compatible interactions (cultivar Zhnitsa) the degree of hormone accumulation was very high (Figure 4). The less intense accumulation of ABA content and less extensive damaged areas in the cv. Kaz10 can be explained by the absence of a second compatible interaction of SnToxA–*Tsn1* in these plants, in contrast to the cv. Zhnitsa. In both cultivars, the H_2_O_2_ content decreased and the CAT activity increased (Figure 3), which correlates with the processes of disease development and the formation of damaged zones on the leaves (Figure 1, Table 1). In Phase III (72 hpi), the changes in the endogenous ABA levels in the cultivars were somewhat different (Figure 4).

ABA did not accumulate in the resistant cv. Om35 or in the susceptible cv. Kaz10 (SnTox3–*Snn3-B1*), but the hormone content significantly increased in the susceptible cv. Zhnitsa (SnToxA–*Tsn1* and SnTox3–*Snn3-B1*) in Phase III (Figure 4). At the same time, H_2_O_2_ generation was insignificant in all the cultivars, and CAT activity was high in the two susceptible cultivars (Kaz10 and Zhnitsa) (Figure 3). Thus, an earlier and short-term increase in the ABA level in the resistant genotype Om35 (Phase II) is necessary for the activation of plant defense responses, and the long-term accumulation of this phytohormone in the susceptible genotype Zhnitsa (Phase II and Phase III) negatively affects plant resistance against the *S. nodorum* SnB isolate. The same pattern of change in the endogenous ABA level was observed in wheat genotypes resistant and susceptible to *Tilletia caries* [43].

The increase in the endogenous ABA level in the leaves of the wheat plants infected with the *S. nodorum* SnB isolate occurred due to the activation of the synthesis of this phytohormone in the plants, and a decrease in the ABA content was achieved, among other things, by inhibiting synthesis, since we observed a positive correlation between the level of the transcripts of the ABA synthesis genes *TaNCED* and *TaZEP* and the endogenous ABA content in the leaves of infected plants (Figure 4 and Figure 5). The same correlation was observed in flax seedlings infected with *Fusarium culmorum* [23]. In addition, infection with the necrotrophic pathogen *B. cinerea* promoted ABA biosynthesis by enhancing transcription by the *NCED3* and *NCED5* genes in Arabidopsis [15].

The effect of exogenous ABA on the development of the defense reactions of wheat against SnB also depended on the presence of compatible interactions, especially in Phase II. In the absence of a compatible interaction in the resistant cv. Om35, the exogenous ABA did not affect the endogenous content of the hormone or the redox status of the plants in Phase II (Figure 3 and Figure 4). In the susceptible cultivars Kaz10 and Zhnitsa, the exogenous ABA increased the endogenous content of the hormone, but had the opposite effect on the redox status of the plants in Phase II (Figure 3 and Figure 4). In the presence of a compatible interaction of SnTox3–*Snn3-B1* in the cv. Kaz10, the exogenous ABA enhanced plant resistance by increasing H_2_O_2_ generation and decreasing CAT activity in Phase II (Figure 1 and Figure 3). In contrast, in the presence of the compatible interactions of SnToxA–*Tsn1* and SnTox3–*Snn3-B1* in the cv. Zhnitsa, the exogenous ABA enhanced plant susceptibility by decreasing H_2_O_2_ generation and increasing CAT activity in Phase II (Figure 1 and Figure 3). In Phase III, the exogenous ABA increased the endogenous hormone content in all three cultivars (Figure 4), but its effect on plant redox status remained the same as in Phase II (Figure 3).

Other studies have shown that ABA can both suppress H_2_O_2_ accumulation and induce it in plants under a pathogen attack [20,23,44]. ABA treatment reduced ROS production and the resistance of tomato plants against *B. cinerea* [44]. In contrast, the ABA-induced resistance of rice against *C. miyabeanus* was based on limiting pathogen growth in the leaf mesophyll, including through rapid H_2_O_2_ accumulation [20]. In flax plants infected with *F. culmorum*, the most affected genes were connected with ROS production and processing, callose synthesis, and ABA production [23]. The role of ABA in regulating the work of CAT has not been sufficiently studied; the data found in the literature are few and segmental, but some studies have shown that ABA is able to activate CAT [45]. *TaNAC29*-overexpressing plants have showed an ABA-hypersensitive response and increased levels of CAT activity [46]. We assume that the positive or negative role of ABA in the development of plant resistance against a pathogen depends on the presence of a particular NE.

Thus, in the absence of compatible interactions and in the presence of an SnTox3–*Snn3-B1* interaction, ABA induces wheat resistance against SnB by affecting the redox status of plants, and in the presence of an SnToxA–*Tsn1* interaction, ABA increases plant susceptibility to a pathogen by suppressing ROS accumulation. Thus, it can be assumed that the compatible interaction of SnToxA-*Tsn1* present in the cv. Zhnitsa played a negative role in the effect of ABA on the plant’s defense system or, in other words, the *S. nodorum* SnB isolate by the NE SnToxA manipulated the ABA signaling pathway for its growth and development. This hypothesis is supported by our results for the expression of the NEs *SnTox3* and *SnToxA* genes during an infection of the various wheat genotypes (Figure 2). The expression of the *SnTox3* gene does not depend on the wheat genotype, which is consistent with the results of other researchers [27]. And the treatment of plants with ABA does not affect or decrease the expression of this gene, with the exception of Phase III in the cv. Zhnitsa (Figure 2). The inhibition of the expression of the *SnTox3* gene suggests a decrease in SnB virulence, since a positive correlation has been shown between the expression level of NE genes and the degree of virulence of a strain [31,47]. In contrast, the expression of the *SnToxA* gene depends on the wheat genotype and is higher in the presence of a compatible interaction of SnToxA–*Tsn1*, which is consistent with the literature data [47]. The treatment of plants with ABA significantly increases the expression of the *SnToxA* gene during an infection of the cv. Zhnitsa in the presence of a compatible interaction of SnToxA–*Tsn1*, which suggests an increase in the virulence of the *S. nodorum* SnB isolate. This hypothesis is also supported by the results obtained from our previous study of the effect of ABA on the growth and sporulation of the *S. nodorum* SnB isolate in vitro [48]. The correlation analysis showed that ABA could increase the virulence of the SnB isolate associated with the expression of the *SnToxA* gene and reduce the virulence associated with the expression of the *SnTox3* gene [48]. In addition, we showed that the S*. nodorum* SnB isolate produces ABA [48].

Intervention in the phytohormone pathways is a universal strategy adopted by pathogens [4,49]. The *Pseudomonas syringae* effector AvrPtoB increases ABA levels and enhances susceptibility to *P. syringae* by modulating ABA biosynthesis and response pathways in the host plant [50]. Another effector, AvrXccC of *Xanthomonas campestri,* causes ABA accumulation by inducing the ABA biosynthesis gene *NCED5* in Arabidopsis plants, which leads to the increased virulence of the pathogen [51]. On the contrary, the effector protein EqCSEP01276 of the powdery mildew fungus *Erysiphe quercicola* inhibits ABA biosynthesis in Rubber trees (*Hevea brasiliensis*) by affecting *HbNCED5*, thereby inhibiting host defense [52].

Thus, the positive or negative effect of ABA on plant resistance to a pathogen may be associated with the activity of a specific effector that uses the ABA signaling pathway for its own purposes. To identify the putative mechanism of action of the SnToxA effector on the ABA signaling pathway in wheat plants, we studied the expression of some ABA signaling genes. The expression of the *TaRCAR* receptor gene increased in all the cultivars regardless of the genotype (Figure 6A), suggesting an increase in plant sensitivity to ABA [53]. However, sensitivity to the phytohormone ABA in the early stages of infection with the pathogen *S. nodorum* increased to a greater extent in the resistant cv. Om35, and the susceptible cultivars Kaz10 and Zhnitsa had less sensitivity to ABA (Figure 6A). The data on cereal ABA receptors are limited, but the wheat ABA receptor TaPYL4AS-A has been shown to be involved in modulating plant sensitivity to *F. graminearum* [53].

The expression of the *TaSnRK2.10* gene, a positive regulator of ABA signaling, also increased in all the cultivars regardless of the genotype in Phase II (Figure 6B), which also suggests an increase in plant sensitivity to ABA [35]. SnRK2 phosphorylates the relevant substrates (transcription factors and proteins), leading to ABA-related physiological responses [16]. A distinctive feature of the effect of exogenous ABA on the expression of the *TaSnRK2.10* gene was the increase in the transcript level of this gene in the cv. Zhnitsa in the presence of a compatible interaction of SnToxA–*Tsn1* at 72 hpi in Phase III (Figure 6B), which could be associated with the development of the increased susceptibility of this cultivar when treated with ABA. We did not find such an effect of ABA on the expression of the *TaSnRK2.10* gene in Phase III in the cultivars Om35 and Kaz10 (Figure 6B). Unfortunately, research on wheat SnRK2 is very limited, especially on the response to biotic stress [35]. The ectopic expression of *TaSnRK2.10* in rice (*Oryza sativa*) conferred drought tolerance [35]. TaSnRK2.10 interacted with and phosphorylated TaERD15 and enolase 1 (TaENO1) in vivo and in vitro. TaERD15 phosphorylated by TaSnRK2.10 was prone to degradation by the 26S proteasome, thereby mitigating its negative effects on drought tolerance [35]. The expression of ten identified wheat *TaSnRK2* genes was enhanced by a water deficit and salt and cold stresses, where the water stress had the greatest effect on increasing the expression of *TaSnRK2*s [54]. It was shown that SnRK2 may play a key role in the regulation of ABA-responsive genes through the phosphorylation of substrates such as ABI5 in the response of cereals to various stresses [16]. The mechanism of action of the conserved *Pseudomonas* effector AvrPtoB has recently been elucidated, acting as an E3 ubiquitin ligase and promoting bacterial virulence [55]. SnRK2.8, which interacts with AvrPtoB in yeast and in planta, was required for AvrPtoB virulence functions, including the suppression of callose deposition and the targeting of the plant defense regulator NPR1 [55]. Thus, we assume that, depending on the presence of the SnToxA effector, TaSnRK2.10 can activate various substrates and promote resistance or susceptibility to the pathogen *S. nodorum*.

The analysis of the expression of the *TaERD15* and *TaABI5* genes, which are positive regulators of early post-invasive defense and substrates of the *TaSnRK2* genes [37], showed an increase in the level of the transcripts of these genes in the resistant genotype Om35 in the absence of compatible interactions, and in the Kaz10 genotype with one compatible interaction of SnTox3–*Snn3-B1* in Phase II (Figure 6C,D). However, the degree of induction of the expression of the *TaERD15* and *TaABI5* genes in the cv. Kaz10 was lower than in the cultivar Om35 (Figure 6C,D). In the susceptible cv. Zhnitsa, in the presence of the two compatible interactions of SnToxA–*Tsn1* and SnTox3–*Snn3-B1*, no significant induction of the transcription of the *TaERD15* and *TaABI5* genes was observed in Phase II, except for a transient increase in the mRNA content of the *TaERD15* gene at 6 hpi (Figure 6C,D). Thus, based on our results, we suggest that the *TaERD15* and *TaABI5* genes are associated with the development of resistance in wheat plants to the *S. nodorum* isolate SnB.

Many target genes are regulated by ABI5 and the regulation of ABI5 expression is complex and is mediated through many regulators [38]. A recent study uncovered a new role for ABI5 in the phase changes in vegetative growth in plants’ juvenile-to-adult transition associated with miR156 [56]. Increased levels of ABI5 promote the expression of miR156 to keep plants in the juvenile phase because juvenile plants are capable of a higher degree of plasticity and are more resistant to stresses [56]. In addition, our previous and current results demonstrate that the transcript accumulation of *TaABI5* and miR156 shows similar expression patterns in the three studied wheat genotypes, Om35, Kaz10, and Zhnitsa, infected with *S. nodorum* SnB isolate [57]. In addition, an increase in miR156 expression in rice is associated with the development of resistance to *Rhizoctonia solani* [58]. Thus, we assume that the effect of *TaABI5* on wheat plant resistance to *S. nodorum* is associated with the regulation of miR156 expression. It was previously shown that the early ABA-responsive gene ERD15 stimulates resistance against the bacterium *Erwinia carotovora* but suppresses ABA-dependent tolerance to osmotic stress [36]. Although the exact nature of ERD15-dependent resistance is unknown, it seems evident that ERD15 has a role in the crosstalk between biotic and abiotic stress resistance [35].

The analysis of the expression of the *TaNAC29* gene showed an increase in the transcript level of the gene in the susceptible genotypes Kaz10 and Zhnitsa in Phase II, and additionally in the cv. Zhnitsa in Phase III (Figure 7A). The expression pattern of the *TaNAC29* gene in the cv. Zhnitsa coincided with the expression pattern of the *SnToxA* gene (Figure 2 and Figure 7). The results of the transcription analysis of the *TaNAC29* gene obtained with the exogenous addition of ABA showed that the *TaNAC29* gene is an ABA-induced TF, but in *S. nodorum*-infected plants, the induction of this gene expression depended on the presence of the SnToxA effector and the compatible interaction of SnToxA–*Tsn1* (Figure 7A). These results indicate that the TF TaNAC29 is associated with the development of wheat susceptibility to *S. nodorum* SnB isolate. The expression pattern of the *TaNAC29* gene is similar to the changes in CAT activity (Figure 3A). It was previously shown that *TaNAC29*-overexpressed plants exhibit an ABA-hypersensitive response and accumulate lesser amounts of malondialdehyde and H_2_O_2_, while they have higher superoxide dismutase and catalase activities under high salinity and/or dehydration stress [46].

The expression of the *TaNAC21/22* gene was strongly suppressed in the resistant genotype Om35, was not activated in the cv. Kaz10, and was induced in the cv. Zhnitsa only in Phase III (Figure 7B). The results of the transcription analysis of the *TaNAC21/22* gene obtained with the exogenous ABA addition show that *TaNAC21/22* is an ABA-induced and SnToxA-induced TF and is associated with the development of wheat susceptibility to *S. nodorum* SnB isolate (Figure 7B), in particular with a decrease in H_2_O_2_ content, as shown in our work and under drought conditions on Malus domestica apple plants [59]. In addition, *TaNAC21/22* is a target of miR164 and negatively regulates the resistance of wheat to stripe rust *Puccinia striiformis* f. sp. *tritici* (*Pst*) [39]. In addition, our previous and current results demonstrate that the transcript accumulation of *TaNAC21/22* and miR164 show contrasting divergent expression patterns in the three studied wheat genotypes, Om35, Kaz10, and Zhnitsa, infected with *S. nodorum* SnB isolate [57]. Many pathogen effectors interact with NAC TF to facilitate the infection process [41]. Studies have shown that the soil-borne pathogenic fungus *Verticillium dahliae* secretes an effector protein called Protein elicitor from V. dahliae 1 (PevD1), which enters the nucleus of plants and interacts with the NAC transcription factor ORESARA1 (ORE1). PevD1 promotes leaf senescence and facilitates pathogen infection by stabilizing ORE1 [41]. Thus, we can assume that the *S. nodorum* SnToxA effector influences the expression of the *TaNAC29* and *TaNAC21/22* TF genes to increase plant susceptibility through regulating plant redox status and limiting oxidative bursts [41,46,59].

Thus, the results of this work show that the ABA-signaling *TaERD15* and *TaABI5* genes, which are positive regulators of early post-invasive defense, are associated with the development of wheat plant resistance to *S. nodorum* SnB isolate. And the *TaNAC29* and *TaNAC21/22* SnToxA-mediated TF genes cause the development of susceptibility of wheat plants to *S. nodorum* SnB isolate, associated with the regulation of the redox status of infected plants.

## 4. Materials and Methods

### 4.1. Plant and Fungi Materials

The isolate of the fungus *S. nodorum* SnB containing two NEs genes, SnToxA and SnTox3, in its genome was used in this study [33]. This isolate was obtained from a collection at the Institute of Biochemistry and Genetics, Ufa Federal Research Centre, Russian Academy of Sciences, Ufa, Russia (https://ibg.anrb.ru/naychnaya-deyatelnost/bioresyrs/kollekciyasimbioticheskix-mikroorganizmov/ (accessed on 25 November 2024)). Three cultivars of bread spring wheat (*Triticum aestivum* L.) from the Institute of Biochemistry and Genetics Ufa Federal Research Center of the Russian Academy of Sciences (Ufa, Russia) were used in the work. Kazahstanskaya 10 (Kaz10), Omskaya 35 (Om35), and Zhnitsa (Zhn) are modern commercial cultivars, which carry a diverse set of sensitivity genes to *S. nodorum* effectors [34].

### 4.2. Experimental Design

All experimental plants were grown hydroponically on 10% Hoagland–Arnon nutrient medium for seven days under controlled conditions in a KBW E6 plant growth chamber (Binder GmbH, TuBlingen, Germany), as described previously [33,34]. The plants were grown in insulated plastic containers with 70–80 seedlings for each treatment option. Experiments to assess the resistance/susceptibility of three wheat cultivars were carried out on the separated first leaves of 7-day-old seedlings placed in Petri dishes on wet cotton wool containing 0.004% benzimidazole (12–14 leaves/dish) [33]. Experiments to study the expression of genes were performed on intact 7-day-old seedlings.

ABA treatment: The phytohormone ABA (Merck KGaA, Sigma-Aldrich, Darmstadt, Germany), at a final concentration of 2 μM, was added to the Hoagland–Arnon nutrient medium in containers with intact 7-day-old seedlings or to a benzimidazole solution in Petri dishes with separated leaves 24 h before infection with *S. nodorum*. After 24 h, the Hoagland–Arnon medium or benzimidazole solution was replaced with new ones without ABA and the plants were infected with the SnB isolate.

Infection with the pathogen: The leaves of wheat plants were infected with a suspension of spores of the SnB isolate at a concentration of 1 × 10^6^ spores mL^−1^, which was obtained from the sporo-mycelial mass from barley grains by soaking in sterile distilled water [34]. Intact 7-day-old seedlings were sprayed with a spore suspension of *S. nodorum* with the addition of wetting agent Tween-20 (0.02%), and the drops of the fungal spore suspension were applied to the separated leaves in the amount of 5 µL per leaf. Control seedlings or separated leaves were sprayed or dripped with water with the addition of wetting agent Tween-20 (0.02%). Then, the vessels with seedlings and Petri dishes with the separated leaves were closed with caps and transferred to the controlled conditions of the growth chamber [29]. The observation of the development of SNB symptoms and fixation and measurement of damage zones were performed, as described previously [29,34].

### 4.3. Analysis of Hydrogen Peroxide Content and Catalase Activity

The plant material (1:5 weight/volume), approximately 200 mg per one biological replication at 6, 24, and 72 h after inoculation with the *S. nodorum* isolate SnB, was homogenized in 0.05 M solution of Na–phosphate buffer (PB), pH 6.2, and incubated at 4 °C for 30 min. The extract was separated by 15 min of centrifugation at 14,000 RPM in a micro centrifuge (5415 K Eppendorf, Hamburg, Germany) and then the supernatant was analyzed. The H_2_O_2_ concentration in the supernatant was determined by xylenol orange, in the presence of Fe^2+^, where hydroperoxides were reduced by ferrous ions in acid solution forming a ferric product–xylenol orange complex, detected spectrophotometrically at 560 nm [60]. CAT activity was determined using the standard method based on the ability of H_2_O_2_ to form a stable-colored complex with molybdate salts, as described previously [61]. The CAT activity was detected by a microassay in 96-well plates (Corning-Costar, Glendale, AZ, USA) by mixing 150 µL 0.03% H_2_O_2_ (or water as a control) with 20 µL supernatant. The reaction was stopped by the addition of 75 µL 4% ammonium molybdate after 1 min. Optical density was measured at 405 nm on a Benchmark Microplate Reader spectrophotometer (Bio-Rad Laboratories, Hercules, CA, USA). CAT activity was calculated using a calibration curve and expressed in µM H_2_O_2_/(mg protein per min). Protein content was determined by the Bradford method.

### 4.4. Enzyme-Linked Immunosorbent Assay (ELISA) of ABA Content in Wheat Plants

Shoots from seven wheat plants (approximately 500 mg) per one biological replication at 6, 24, and 72 h after inoculation with the *S. nodorum* isolate SnB were homogenized and phytohormone ABA was extracted with 80% ethanol (1:10, weight/volume) for 16 h at 4 °C. The extract was separated by 20 min of centrifugation at 5000 RPM in an Avanti J-E centrifuge (Bekman Coulter, Bray, CA, USA) and evaporated to obtain aqueous residue. ABA from supernatant of the aqueous residue of plant material was extracted with diethyl ether, according to a modified scheme [62]. The extraction was performed with diethyl ether from the aqueous residue, which was acidified with HCl followed by extraction into a sodium bicarbonate solution and re-extraction into diethyl ether (after acidification of sodium bicarbonate), with a decrease in volume at each stage of extraction–re-extraction, after which the samples were methylated with diazomethane [62]. An ABA quantitative assay was performed with ELISA using specific antibodies, as described previously [63]. The reliability of the phytohormone immunoassay was confirmed using a dilution test and through a comparison with the data obtained from the results of high-performance liquid chromatography (HPLC) in combination with mass spectrometry [64,65].

### 4.5. Gene Expression Analysis

To study plant or fungal genes expression, shoots of intact wheat seedlings subjected to various treatments were fixed in liquid nitrogen at 6, 24, and 72 h after inoculation with the *S. nodorum* isolate SnB. Total wheat RNA and total fungal RNA were extracted using Lira^®^ (Biolabmix, Moscow, Russia) according to the manufacturer’s instructions. For cDNA synthesis, the method described in an earlier work was used [66]. Primers for real-time polymerase chain reaction (real-time PCR) were devised using the web tool PrimerQuest™ (https://www.idtdna.com/pages/tools/primerquest (accessed on 25 November 2024)) (Integrated DNA Technologies, Inc., Coralville, IA, USA). The sequences of primers for plant genes encoding enzymes of ABA biosynthesis, the TFs, and proteins of ABA signaling pathway are presented in Table S1 (Supplementary Materials), and the sequences of primers for fungal genes encoding NEs (*SnToxA*, S*nTox3*) are presented in Table S2 (Supplementary Materials). The annealing temperature of the primers was 60 °C. A melting curve analysis was conducted to determine the specificity of the reaction (at 95 °C for 15 s, 60 °C for 1 min, and 95 °C for 15 s). The efficiency of the primers was determined using a series of cDNA dilutions (10-fold). To normalize the results of the expression of the studied wheat genes, primers to the genes of the constitutively expressed proteins RNase L inhibitor-like (*TaRLI*) and glyceraldehyde 3-phosphate dehydrogenase (*TaGAPDH*) were used as an internal control (Table S1 Supplementary Materials). To normalize the results of the expression of the fungal genes, primers to the constitutively expressed *Snβ-tubulin* gene of *S. nodorum* were used (Table S2 Supplementary Materials). Real-time PCR was performed on a “DNA amplifier in real time” CFX96 Touch with fluorescent detection (BioRad Laboratories, Hercules, CA, USA). For detection, a set of reagents, EvaGreen I (Synthol, Moscow, Russia), was used. In order to quantify the relative gene expression, the 2^−ΔΔCT^ method was performed as described earlier [67]. Three independent biological and five technical replications were performed for each experiment.

### 4.6. Statistical Analysis

All experiments were repeated three times with three biological repetitions. Experimental data were expressed as means ± SE, which were calculated for all treatments using Microsoft Excel (version 16.0.14430.20306, Redmond, WA, USA). The significance of differences was assessed by ANOVA followed by Duncan’s test (*p* ≤ 0.05) with STATISTICA 10.0 software (version STA999K347150-W, Tulsa, OK, USA). Before statistical analysis, the normal distribution of all parameters was analyzed and confirmed by the Shapiro–Wilk W test. The treatment variants and the number of repetitions are indicated in the tables and figures.

## 5. Conclusions

ABA is involved in the crosstalk between biotic and abiotic stress responses. Therefore, it represents a critical player in the interplay between abiotic and biotic stress signaling, which will be crucial for breeding crop species with improved abiotic stress tolerance and pathogen resistance [16,37].

**Figure 8:**
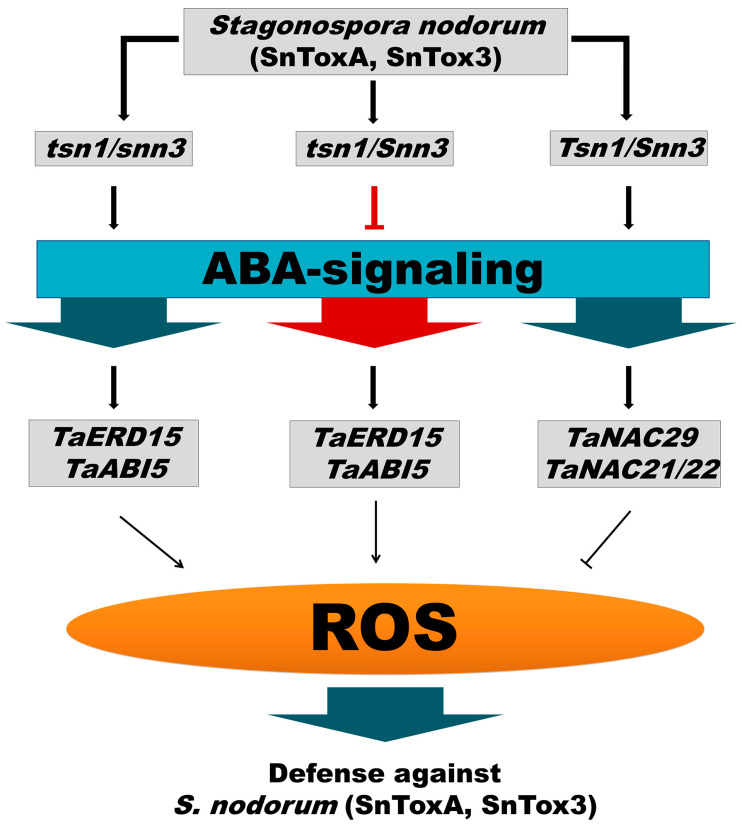
Model illustrating how NEs can manipulate the ABA signaling pathway. The black lines with arrows represent stimulatory effects, and the blunt red and black lines represent inhibitory effects. Designations: *S. nodorum* (SnToxA/SnTox3) is an isolate of the pathogen producing NE SnToxA and SnTox3; *tsn1/snn3*—the wheat genotype has recessive alleles for both susceptibility genes, so it is insensitive to SnToxA and SnTox3; *tsn1/Snn3*—the wheat genotype has recessive allele only for the *tsn1* gene, so it is sensitive only to NE SnTox3; *Tsn1/Snn3*—the wheat genotype has dominant alleles for both susceptibility genes, so it is sensitive to SnToxA and SnTox3; *TaERD15 / TaABI5* activation of ABA signaling occurs through the induction of the expression of the *TaERD15* and *TaABI5* genes; *TaNAC29*/*TaNAC21/22* activation of ABA signaling occurs through the induction of the expression of the *TaNAC29* and *TaNAC21/22* genes.

Taken together, the results of this work on the gene expression and redox status of resistant and sensitive wheat genotypes treated with ABA show that ABA signaling is directed at inducing the resistance of wheat plants to *S. nodorum* SnB isolate through the activation of the early post-invasive defense genes *TaERD15* and *TaABI5.* The Induction of the expression of these genes leads to ROS accumulation in the early stage of infection, with the subsequent limitation of pathogen growth (Figure 8).

The *S. nodorum* NEs SnToxA and SnTox3 have different effects on ABA signaling. In the presence of a compatible interaction of SnTox3–*Snn3-B1*, ABA signaling is suppressed, most likely indirectly through the activation of another hormonal signaling pathway (Figure 8). Based on our previous results, it can be concluded that the ethylene signaling pathway activated by SnTox3 during an infection of susceptible genotypes suppresses the ABA signaling pathway [29]. Our findings are supported by the results obtained for *C. miyabeanus*-infected rice, where ABA enhanced resistance against *C. miyabeanus* at least in part by suppressing pathogen-induced ethylene action in an OsMPK5-dependent manner [20]. In the presence of a compatible interaction of SnToxA–*Tsn1*, ABA signaling is activated, but the activity of the early post-invasive defense genes *TaERD15* and *TaABI5* is inhibited, and the expression of the NAC family TF genes *TaNAC29* and *TaNAC21/22* is induced. The TF genes *TaNAC29* and *TaNAC21/22* SnToxA-mediately induce the development of the susceptibility of wheat plants to S*. nodorum* SnB, associated with a decrease in the oxidative burst in the early stage of infection (Figure 8).

The NAC TFs are key regulators of plant growth and development, regulators of yield-related traits, and play a key role in plant immunity, being positive or negative modulators [68]. As one of the largest families of plant TFs, the NACs modulate the transcriptional regulatory activity of gene networks, and knowledge of the coregulation of the NACs with other genes could further elucidate the cooperation of gene networks. So, it is relevant and important to study the role and functions of the NAC TFs to improve crop yield and productivity [68].

Thus, our study provides new data on the role of the NEs SnTox3 and SnToxA in manipulating ABA signaling for the development of the susceptibility of wheat to *S. nodorum*. Future studies should focus on further elucidating the molecular mechanisms underlying the crosstalk of ABA signaling with other SA, JA, and ethylene hormone signaling pathways. Deepening our knowledge in this area will be instrumental for developing new strategies for breeding programs and will contribute to the development of environmentally friendly sustainable agriculture.

## Figures and Tables

**Table 1:** The effect of treatment with ABA on the damage areas of leaves of the three wheat genotypes infected with *S. nodorum* SnB.

Variant of Treatment	Cultivars (Genotypes)		
	Omskaya 35 (*tsn1/snn3*)	Kazakhstanskaya 10 (*tsn1/Snn3*)	Zhnitsa (*Tsn1/Snn3*)
*S. nodorum* (SnToxA/SnTox3) *	8 ± 0.5 a	61 ± 4 b	81 ± 5 d
*S. nodorum* + Abscisic acid	9 ± 0.4 a	29 ± 2 c	97 ± 6 e

* The leaf area and lesion area were measured 8 days after infection with the pathogen isolate. The area of the damage zones is presented as a percentage of the total leaf area, which is taken as 100%. The values are expressed as the mean SE (*n* = 30) (ANOVA with Duncan’s test; comparisons between genotypes are presented; significant differences are marked with different letters at *p* ≤ 0.05).

## Data Availability

Data are contained within the article and supplementary files.

## Supplementary Materials

The following supporting information can be downloaded at https://www.mdpi.com/article/10.3390/plants14030355/s1, Table S1: The sequences of wheat primers used for real-time PCR; Table S2: The sequences of fungi primers used for real-time PCR.

## References

[1] Jones J.D.G., Dang J.L. (2006). The plant immune system. Nature.

[2] Ngou B.P.M., Ding P., Jones J.D.G. (2022). Thirty years of resistance: Zig-zag through the plant immune system. Plant Cell.

[3] Tan K.C., Oliver R.P., Solomon P.S., Moffat C.S. (2010). Proteinaceous necrotrophic effectors in fungal virulence. Funct. Plant Biol..

[4] Zhang S., Li C., Si J., Han Z., Chen D. (2022). Action Mechanisms of Effectors in Plant-Pathogen Interaction. Int. J. Mol. Sci..

[5] Jung H.W., Panigrahi G.K., Jung G.Y., Lee Y.J., Shin K.H., Sahoo A., Choi E.S., Lee E., Kim K.M., Yang S.H. (2020). Pathogen-associated molecular pattern-triggered immunity involves proteolytic degradation of core nonsense-mediated mRNA decay factors during the early defense response. Plant Cell.

[6] Yuan M., Ngou B.P.M., Ding P., Xin X.F. (2021). PTI-ETI crosstalk: An integrative view of plant immunity. Curr. Opin. Plant Biol..

[7] Kim C.-Y., Song H., Lee Y.-H. (2022). Ambivalent response in pathogen defense: A double-edged sword?. Plant Comm..

[8] Liu Z., Zhang Z., Faris J.D., Oliver R.P., Syme R., McDonald M.C., McDonald B.A., Solomon P.S., Lu S., Shelver W.L. (2012). The cysteine rich necrotrophic effector SnTox1 produced by *Stagonospora nodorum* triggers susceptibility of wheat lines harboring *Snn1*. PLoS Pathog..

[9] Winterberg B., Du Fall A.L., Song X., Pascovici D., Care N., Molloy M., Ohms S., Solomon P.S. (2014). The necrotrophic effector protein SnTox3 re-programs metabolism and elicits a strong defence response in susceptible wheat leaves. BMC Plant Biol..

[10] Shi G., Zhang Z., Friesen T.L., Raats D., Fahima T., Brueggeman R.S., Lu S., Trick H.N., Liu Z., Chao W. (2016). The hijacking of a receptor kinase–driven pathway by a wheat fungal pathogen leads to disease. Sci. Adv..

[11] Ali S., Tyagi A., Mir Z.A. (2024). Plant Immunity: At the Crossroads of Pathogen Perception and Defense Response. Plants.

[12] Robert-Seilaniantz A., Grantm M., Jones J.D. (2011). Hormone crosstalk in plant disease and defense: More than just jasmonate-salicylate antagonism. Annu. Rev. Phytopathol..

[13] Wang Y., Mostafa S., Zeng W., Jin B. (2021). Function and mechanism of jasmonic acid in plant responses to abiotic and biotic stresses. Int. J. Mol. Sci..

[14] Spoel S.H., Dong X. (2024). Salicylic acid in plant immunity and beyond. Plant Cell.

[15] Chen K., Li G.-J., Bressan R.A., Song C.-P., Zhu J.-K., Zhao Y. (2020). Abscisic acid dynamics, signaling, and functions in plants. J. Integr. Plant Biol..

[16] Gietler M., Fidler J., Labudda M., Nykiel M. (2020). Abscisic Acid—Enemy or Savior in the Response of Cereals to Abiotic and Biotic Stresses?. Int. J. Mol. Sci..

[17] Hewage K.A.H., Yang J.-F., Wang D., Hao G.-F., Yang G.-F., Zhu J.-K. (2020). Chemical Manipulation of Abscisic Acid Signaling: A New Approach to Abiotic and Biotic Stress Management in Agriculture. Adv. Sci..

[18] Maksimov I.V. (2009). Abscisic Acid in the Plants–Pathogen Interaction. Russ. J. Plant Physiol..

[19] Ton J., Mauch-Mani B. (2004). Beta-amino-butyric acid-induced resistance against necrotrophic pathogens is based on ABA-dependent priming for callose. Plant J..

[20] De Vleesschauwer D., Yang Y., Cruz C.V., Hofte M. (2010). Abscisic acid-induced resistance against the brown spot pathogen *Cochliobolus miyabeanus* in rice involves MAPK-mediated repression of ethylene signaling. Physiol. Plant..

[21] Spence C.A., Lakshmanan V., Donofrio N., Bais H.P. (2015). Crucial Roles of Abscisic Acid Biogenesis in Virulence of Rice Blast Fungus *Magnaporthe oryzae*. Front. Plant Sci..

[22] Han X., Kahmann R. (2019). Manipulation of phytohormone pathways by effectors of filamentous plant pathogens. Front. Plant Sci..

[23] Boba A., Kostyn K., Kochneva Y., Wojtasik W., Mierziak J., Prescha A., Augustyniak B., Grajzer M., Szopa J., Kulma A. (2022). Abscisic Acid—Defensive Player in Flax Response to *Fusarium culmorum* Infection. Molecules.

[24] Adie B., Chico J.M., Rubio-Somoza I., Solano R. (2007). Modulation of Plant Defenses by Ethylene. J. Plant Growth Regul..

[25] Haugrud A.R.P., Zhang Z., Friesen T.L., Faris J.D. (2022). Genetics of resistance to *septoria nodorum* blotch in wheat. Theor. Appl. Genet..

[26] McDonald M.C., Solomon P.S. (2018). Just the surface: Advances in the discovery and characterization of necrotrophic wheat effectors. Curr. Opin. Microbiol..

[27] Phan H.T.T., Rybak K., Furuki E., Breen S., Solomon P.S., Oliver R.P., Tan K.C. (2016). Differential effector gene expression underpins epistasis in a plant fungal disease. Plant J..

[28] Breen S., Williams S.J., Winterberg B., Kobe B., Solomon P.S. (2016). Wheat PR-1 proteins are targeted by necrotrophic pathogen effector proteins. Plant J..

[29] Veselova S.V., Nuzhnaya T.V., Burkhanova G.F., Rumyantsev S.D., Khusnutdinova E.K., Maksimov I.V. (2021). Ethylene-cytokinin interaction determines early defense response of wheat against *Stagonospora nodorum* Berk. Biomolecules.

[30] Veselova S., Nuzhnaya T., Maksimov I. (2024). The Role of Salicylic, Jasmonic Acid and Ethylene in the Development of the Resistance/Susceptibility of Wheat to the SnTox1-Producing Isolate of the Pathogenic Fungus *Stagonospora nodorum* (Berk.). Plants.

[31] Virdi S.K., Liu Z., Overlander M.E., Zhang Z., Xu S.S., Friesen T.L., Faris J.D. (2016). New Insights into the Roles of Host Gene-Necrotrophic Effector Interactions in Governing Susceptibility of Durum Wheat to Tan Spot and *Septoria Nodorum* Blotch. G3 (Bethesda).

[32] Faris J.D., Zhang Z., Lu H., Lu S., Reddy L., Cloutier S., Fellers J.P., Meinhardt S.W., Rasmussen J.B., Xu S.S. (2010). A unique wheat disease resistance-like gene governs effector-triggered susceptibility to necrotrophic pathogens. Proc. Natl. Acad. Sci. USA.

[33] Veselova S., Nuzhnaya T., Burkhanova G., Rumyantsev S., Maksimov I. (2021). Reactive oxygen species in host plant are required for an early defense response against attack of *Stagonospora nodorum* Berk. necrotrophic effectors SnTox. Plants.

[34] Nuzhnaya T., Veselova S., Burkhanova G., Rumyantsev S., Shoeva O., Shein M., Maksimov I. (2023). Novel sources of resistance to *Stagonospora nodorum* and role of effector-susceptibility gene interactions in wheat of russian breeding. Int. J. Plant Biol..

[35] Zhang Y., Wang J., Li Y., Zhang Z., Yang L., Wang M., Zhang Y., Zhang J., Li C., Li L. (2023). Wheat TaSnRK2.10 phosphorylates TaERD15 and TaENO1 and confers drought tolerance when overexpressed in rice. Plant Physiol..

[36] Kariola T., Brader G., Helenius E., Li J., Heino P., Palva E.T. (2006). Early responsive to dehydration 15, a negative regulator of abscisic acid responses in Arabidopsis. Plant Physiol..

[37] Ton J., Flors V., Mauch-Mani B. (2009). The multifaceted role of ABA in disease resistance. Trends Plant Sci..

[38] Skubacz A., Daszkowska-Golec A., Szarejko I. (2016). The Role and Regulation of ABI5 (ABA-Insensitive 5) in Plant Development, Abiotic Stress Responses and Phytohormone Crosstalk. Front. Plant Sci..

[39] Feng H., Duan X., Zhang Q., Li X., Wang B., Huang L., Wang X., Kang Z. (2014). The target gene of tae-miR164, a novel NAC transcription factor from the NAM subfamily, negatively regulates resistance of wheat to stripe rust. Mol. Plant Pathol..

[40] Lv S., Guo H., Zhang M., Wang Q., Zhang H., Ji W. (2020). Large-Scale Cloning and Comparative Analysis of TaNAC Genes in Response to Stripe Rust and Powdery Mildew in Wheat (*Triticum aestivum* L.). Genes.

[41] Dong B., Liu Y., Huang G., Song A., Chen S., Jiang J., Chen F., Fang W. (2024). Plant NAC transcription factors in the battle against pathogens. BMC Plant Biol..

[42] Cao F.Y., Yoshioka K., Desveaux D. (2011). The roles of ABA in plant–pathogen interactions. J. Plant Res..

[43] Maksimov I.V., Ganiev R.M., Khairullin R.M. (2002). Changes in the levels of IAA, ABA, and cytokinins in wheat seedlings infected with *Tilletia caries*. Russ. J. Plant Physiol..

[44] Sivakumaran A., Akinyemi A., Mandon J., Cristescu S.M., Hall M.A., Harren F.J.M., Mur L.A.J. (2016). ABA Suppresses *Botrytis cinerea* Elicited NO Production in Tomato to Influence H_2_O_2_ Generation and Increase Host Susceptibility. Front. Plant Sci..

[45] Wang W.-R., Liang J.-H., Wang G.-F., Sun M.-X., Peng F.-T., Xiao Y.-S. (2020). Overexpression of *PpSnRK1α* in tomato enhanced salt tolerance by regulating ABA signaling pathway and reactive oxygen metabolism. BMC Plant Biol..

[46] Huang Q., Wang Y., Li B., Chang J., Chen M., Li K., Yang G., He G. (2015). TaNAC29, a NAC transcription factor from wheat, enhances salt and drought tolerance in transgenic Arabidopsis. BMC Plant Biol..

[47] Faris J.D., Zhang Z., Rasmussen J.B., Friesen T.L. (2011). Variable expression of the *Stagonospora nodorum* effector SnToxA among isolates is correlated with levels of disease in wheat. Mol. Plant-Microbe Interact..

[48] Nuzhnaya T.V., Sorokan A.V., Burkhanova G.F., Maksimov I.V., Veselova S.V. (2024). The Role of Cytokinins and Abscisic Acid in the Growth, Development and Virulence of the Pathogenic Fungus *Stagonospora nodorum* (Berk.). Biomolecules.

[49] Kazan K., Lyons R. (2014). Intervention of Phytohormone Pathways by Pathogen Effectors. Plant Cell.

[50] de Torres M., Mansfield J.W., Grabov N., Brown I.R., Ammouneh H., Tsiamis G., Forsyth A., Robatzek S., Grant M., Boch J. (2006). *Pseudomonas syringae* effector AvrPtoB suppresses basal defence in Arabidopsis. Plant J..

[51] Ho Y.P., Tan C.M., Li M.Y., Lin H., Deng W.L., Yang J.Y. (2013). The AvrB_AvrC domain of AvrXccC of *Xanthomonas campestris* pv. campestris is required to elicit plant defense responses and manipulate ABA homeostasis. Mol. Plant Microbe Interact..

[52] Li X., Liu Y., He Q., Li S., Liu W., Lin C., Miao W. (2020). A Candidate Secreted Effector Protein of Rubber Tree Powdery Mildew Fungus Contributes to Infection by Regulating Plant ABA Biosynthesis. Front. Microbiol..

[53] Gordon C.S., Rajagopalan N., Risseeuw E.P., Surpin M., Ball F.J., Barber C.J., Buhrow L.M., Clark S.M., Page J.E., Todd C.D. (2016). Characterization of *Triticum aestivum* abscisic acid receptors and a possible role for these in mediating Fusarium head blight susceptibility in wheat. PLoS ONE.

[54] Zhang H., Li W., Mao X., Jing R., Jia H. (2016). Differential activation of the wheat SnRK2 family by abiotic stresses. Front. Plant Sci..

[55] Lei L., Stevens D.M., Coaker G. (2020). Phosphorylation of the Pseudomonas effector AvrPtoB by *Arabidopsis* SnRK2.8 Is Required for Bacterial Virulence. Mol. Plant..

[56] Guo C., Jiang Y., Shi M., Wu X., Wu G. (2021). ABI5 acts downstream of miR159 to delay vegetative phase change in Arabidopsis. New Phytol..

[57] Nuzhnaya T., Veselova S., Burkhanova G., Maksimov I. (2023). Virulence Factors of the Fungal Pathogen *Stagonospora nodorum* Manipulate Hormonal Signaling Pathways in *Triticum aestivum* L. by Regulating Host Plant MicroRNA Expressions. Front. Biosci. (Elite Ed).

[58] Sasani S.T., Soltani B.M., Mehrabi R., Padasht-Dehkaei H.S.F. (2020). Expression alteration of candidate rice miRNAs in response to sheath blight disease. Iran. J. Biotechnol..

[59] Peng X., Feng C., Wang Y.-T., Zhang X., Wang Y.-Y., Sun Y.-T., Xiao Y.-Q., Zhai Z.-F., Zhou X., Du B.-Y. (2022). miR164g-MsNAC022 acts as a novel module mediating drought response by transcriptional regulation of reactive oxygen species scavenging systems in apple. Hortic. Res..

[60] Bindschedler L.V., Dewdney J., Blee K.A. (2006). Peroxidase-dependent apoplastic oxidative burst in Arabidopsis required for pathogen resistance. Plant J..

[61] Rumyantsev S.D., Veselova S.V., Burkhanova G.F., Alekseev V.Y., Maksimov I.V. (2023). *Bacillus subtilis* 26D Triggers Induced Systemic Resistance against *Rhopalosiphum padi* L. by Regulating the Expression of Genes AGO, DCL and microRNA in Bread Spring Wheat. Microorganisms.

[62] Bouizgarne B., Maheshwari D.K. (2012). Bacteria for plant growth promotion and disease management. Bacteria in Agrobiology: Disease Management.

[63] Kudoyarova G.R., Veselov D.S., Sharipova G.V., Akhiyarova R.G., Dodd I.C., Veselov S.Y. (2014). Water relations and growth of original barley plants and its ABA-deficient mutants at increased air temperature. Russ. J. Plant Physiol..

[64] Veselov S.Y., Timergalina L.N., Akhiyarova G.R., Kudoyarova G.R., Korobova A.V., Ivanov I., Arkhipova T.N., Prinsen E. (2018). Study of cytokinin transport from shoots to roots of wheat plants is informed by a novel method of differential localization of free cytokinin bases or their ribosylated forms by means of their specific fixation. Protoplasma.

[65] Kudoyarova G.R., Korobova A.V., Akhiyarova G.R., Arkhipova T.N., Zaytsev D.Y., Prinsen E., Egutkin N.L., Medvedev S.S., Veselov S.Y. (2014). Accumulation of cytokinins in roots and their export to the shoots of durum wheat plants treated with the protonophore carbonyl cyanide m-chlorophenylhydrazone (CCCP). J. Exp. Bot..

[66] Veselova S.V., Sorokan A.V., Burkhanova G.F., Rumyantsev S.D., Cherepanova E.A., Alekseev V.Y., Sarvarova E.R., Kasimova A.R., Maksimov I.V. (2022). By modulating the hormonal balance and ribonuclease activity of tomato plants *Bacillus subtilis* induces defense response against potato virus X and potato Virus Y. Biomolecules.

[67] Schmittgen T.D., Livak K.J. (2008). Analyzing real-time PCR data by the comparative CT method. Nat. Protoc..

[68] Iqbal N., Czékus Z., Poór P., Ördög A. (2023). Ethylene-dependent regulation of oxidative stress in the leaves of fusaric acid-treated tomato plants. Plant Physiol. Biochem..

