# Supplementary material for: Abscisic Acid Can Play a Dual Role in the Triticum aestivum–Stagonospora nodorum Pathosystem
Source: Plants (Basel). 2025 Jan 24;14(3):355. doi: 10.3390/plants14030355 (PMC11820657; doi:10.3390/plants14030355)
Supplement: Supplementary file 1 [file plants-14-00355-s001.zip › plants-3423265-supplementary/Plants-Suppl-Veselova-2024.pdf]

Table S1. The sequences of wheat primers used for real-time PCR

Genes	Strand	5′ to 3′ Primer Sequences	GenBank accession number
TaNCED1	Forward	CCTGCTGCCTCTTCTGCT	KP099105.1
	Reverse	ACCAAGTGCTCTTCCGTCTC	
TaZEP	Forward	TTGGAATGCCTTTGATGC	AF384103.2
	Reverse	GCTGGTTGTTTGCCTTGT	
TaRCAR	Forward	ATCATAACAGTCCACCCACAG	MG273658.1
	Reverse	CACGGCCTCAACGAAGTA	
TaABI5	Forward	CGTGAAGTTCTCTGAGGAAGAAG	AB238932.1
	Reverse	GTCAGCGCGAAGATGGAATA	
TaNAC29	Forward	CACCTACAAGCCCATCAAGT	MN747213.1
	Reverse	GTCTTCTTGTAGATCCGGCATAG	
TaNAC21/22	Forward	CTTCAGTAATAACAGTGCCAGCAA	KC771286.1
	Reverse	ACAGTTCAAGGCGGGGTCTA	
TaSnRK2.10	Forward	CGTGACTTGAAGCTGGAGAA	KJ018723.1
	Reverse	AAGAACCGACGACTTGGAATAG	
TaERD15	Forward	GCGAGCTCTTCTGCTCTTAAT	XM_044495104.1
	Reverse	GTGAACCTCCCTTCGATCTTG	
TaRLI	Forward	TTGAGCAACTCATGGACCAG	AY059462
	Reverse	GCTTTCCAAGGCACAAACAT	
TaGAPDH	Forward	GTGTTCCCACTGTTGATGTTTC	EU022331.1
	Reverse	CCTCCTTGATAGCAGCCTTAAT	

Table S2. The sequences of fungi primers used for real-time PCR

Genes	Strand	5′ to 3′ Primer Sequences	GenBank accession number
SnToxA	Forward	AACGCCAATACAGTGCGAGT	JX997419
	Reverse	GCTGCATTCTCCAATTTTCACG	
	Reverse	CGCTTGTTTGCCGTTCTTAC	
SnTox3	Forward	CGAGCTGATATCCCGTTTGA	FJ823644
	Reverse	GGGACAGTGACAATAGGTAAGG	
Snβ-tubulin	Forward	ACACCAGGAACAACTGCTAACAGC	S56922
	Reverse	TATGCGCGCGTGCTGCAAATTCGA	
